# Rectal metastasis in a patient with long-term breast cancer: a rare case report with literature review

**DOI:** 10.3389/fonc.2026.1597103

**Published:** 2026-01-21

**Authors:** Xiaohong Lyu, Feng Mao

**Affiliations:** Department of Breast Surgery, Peking Union Medical College Hospital, Chinese Academy of Medical Sciences, Peking Union Medical College, Beijing, China

**Keywords:** breast cancer, diagnosis, invasive ductal carcinoma, prognosis, rectal metastasis

## Abstract

Gastrointestinal (GI) metastases from breast cancer are uncommon, with rectal involvement being particularly rare. Here, we present a case of a 55-year-old female with a history of bilateral breast cancer who developed rectal metastasis seven years after initial diagnosis. The patient was initially diagnosed in 2017 with left breast invasive ductal carcinoma (IDC) and right breast ductal carcinoma *in situ* (DCIS). Despite receiving comprehensive treatment, including modified radical mastectomy, chemotherapy, radiotherapy, and endocrine therapy, she experienced multiple metastases involving the bones, lymph nodes, and pleura. In 2024, she presented with new bowel symptoms, and colonoscopy revealed rectal wall thickening with stenosis. Biopsy confirmed metastatic breast cancer with immunohistochemistry showing GATA3(+), CDX2(-), and loss of hormone receptor expression compared to the primary tumor. This case underscores the importance of considering gastrointestinal metastasis in breast cancer patients with bowel symptoms, even years after initial treatment. We also review the literature regarding the literature on the diagnosis, treatment, and prognosis of breast cancer rectal metastasis.

## Introduction

Breast cancer is the most common malignancy in women, with well-documented metastatic patterns primarily to the bones, lungs, liver, and brain (1). Gastrointestinal (GI) tract metastases from breast cancer are relatively rare, occurring in less than 1% of cases, with the stomach being the most frequently involved site (2). Rectal metastasis, in particular, is exceedingly uncommon, and only a limited number of cases have been reported in the literature (3). While invasive lobular carcinoma (ILC) is often associated with a higher propensity for GI metastasis due to its diffuse growth pattern and loss of E-cadherin expression, invasive ductal carcinoma (IDC), the more common subtype of breast cancer, can also metastasize to the GI tract (4, 5). This case highlights the need for clinicians to remain vigilant for rectal metastasis even in cases of IDC, particularly when patients present with non-specific gastrointestinal symptoms that may mimic primary colorectal malignancies (6).

## Case presentation

A 55-year-old female with a family history of breast cancer (her mother was diagnosed in 2007) initially presented in March 2017 with a left breast mass. Imaging studies, including mammography and ultrasound, revealed suspicious lesions in both breasts. Biopsies confirmed left breast invasive carcinoma (IDC) and right breast ductal carcinoma *in situ* (DCIS).

### Initial treatment and pathology

The patient underwent left modified radical mastectomy and right mastectomy with sentinel lymph node biopsy in May 2017. Pathology revealed invasive carcinoma in the left breast (NST, grade 2, size 3.5×2.5×2.3cm) with extensive lymph node involvement (9/14 axillary nodes and 2/6 sentinel nodes positive). Immunohistochemistry (IHC) revealed estrogen receptor (ER) positivity (90%), low Ki-67(11%), and HER2-negative status. The right breast showed low-grade DCIS with clear margins. Postoperative treatment included dose-dense chemotherapy (EC regimen followed by paclitaxel), chest wall radiotherapy, and endocrine therapy (anastrozole with goserelin). The patient initially achieved disease control with this approach.

### Disease progression

In May 2019, bone metastasis was detected (**Table 1**). Despite continued endocrine therapy, progressive disease was noted, with chest wall and lymph node metastases observed in 2021. Biopsy of recurrent lesions revealed reduced ER expression (30%) and maintained HER2-negative status. Treatment was changed to exemestane plus palbociclib with goserelin. Further progression occurred with the development of pleural and pericardial effusions in 2022-2023. Cytology confirmed metastatic breast cancer with complete loss of hormone receptor expression and increased Ki-67 (60-80%). Endocrine therapy was discontinued in October 2022 due to the progression to ER-negative disease. The patient received salvage chemotherapy with docetaxel plus capecitabine (6 cycles), followed by maintenance capecitabine from February 2023.

**Table 1:** Clinical course of the patients with breast cancer (2017-2024).

Timeline	Event	Pathology/IHC	Treatment	Outcome
March 2017	Initial presentation	Left breast: IDC (NST, grade 2, size 3.5×2.5×2.3 cm), ER 90%, Ki-67 11%, HER2 (-). Right breast: DCIS.	Left modified radical mastectomy, right mastectomy with sentinel lymph node biopsy.	Diagnosis confirmed.
May 2017	Post-surgery pathology	Left: IDC with 9/14 axillary and 2/6 sentinel lymph nodes positive. Right: low-grade DCIS with clear margins.	Dose-dense EC → paclitaxel, left chest wall radiotherapy, endocrine therapy (anastrozole + goserelin).	Initial disease control achieved.
May 2019	Bone metastasis detected	N/A	Continued endocrine therapy.	Disease progression noted.
August 2021	Chest wall and lymph node metastases	Reduced ER expression (30%), HER2 (-).	Exemestane + palbociclib + goserelin.	Disease progression continued.
October 2022	Pleural and pericardial effusions	Complete loss of ER/PR, HER2 (1+), Ki-67 60–80%.	Salvage chemotherapy (docetaxel + capecitabine)×6	Disease stabilization achieved temporarily.
February 2023			maintenance capecitabine 1.5g bid, Take orally for 2 weeks, then stop for 2 weeks + Zoledronic acid 4mg q3m	
August 2024	Rectal metastases	GATA3 (+), CDX2 (-), ER/PR (-), HER2 (1+), Ki-67 50%, GCDFP15 (-).	Intestinal stent placement for obstruction relief.	After stent placement, the patient did not return for follow-up care.

IDC, invasive ductal carcinoma; DCIS, ductal carcinoma *in situ*; NST, no special type; ER, estrogen receptor; PR, progesterone receptor; HER2, human epidermal growth factor receptor 2; Ki-67, proliferation index; IHC, immunohistochemistry; EC, epirubicin + cyclophosphamide.

### Rectal metastasis

**Figure 1:**
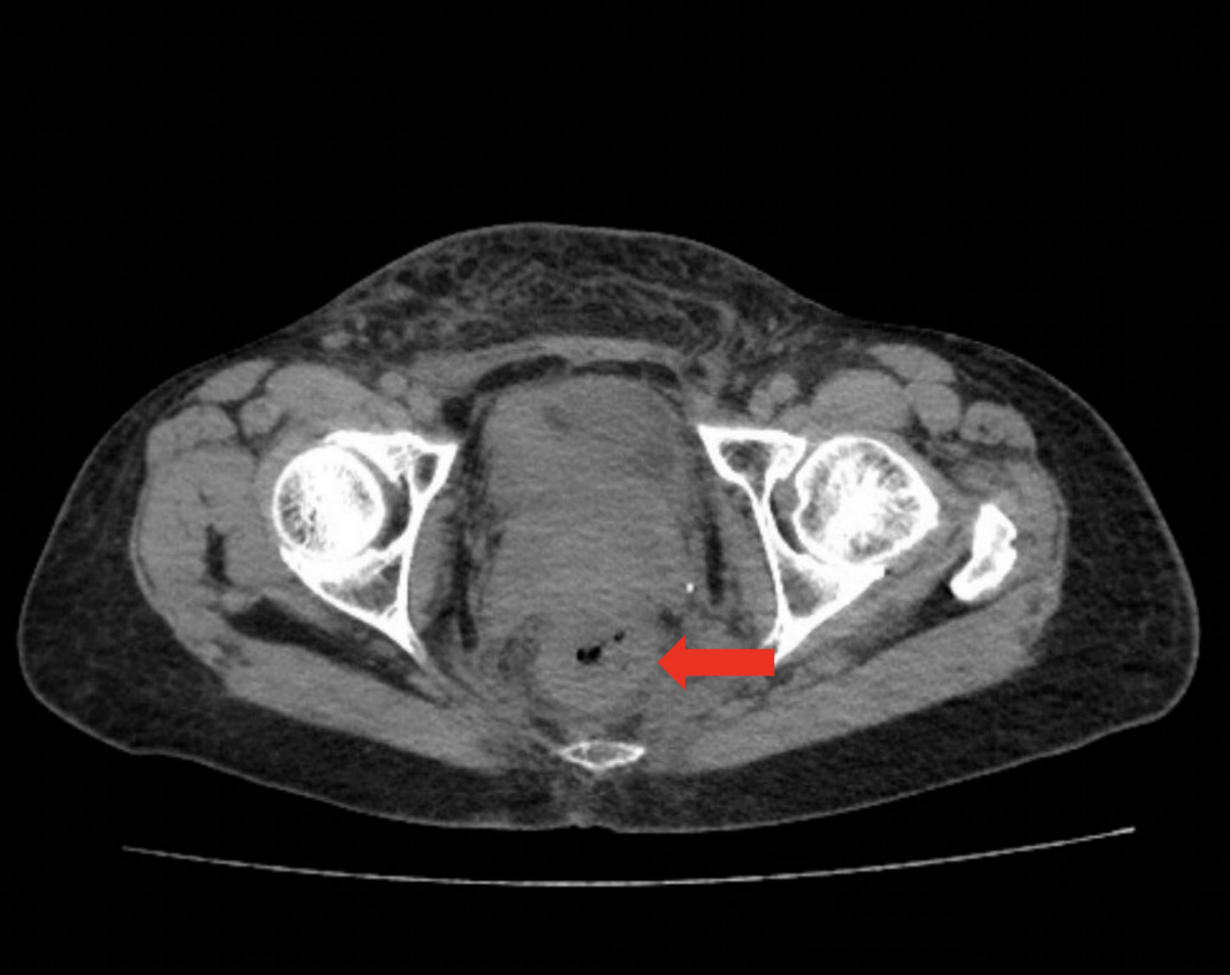
CT imaging of rectal metastasis. Red arrows indicate the site of metastatic involvement in the rectum, characterized by significant thickening of the bowel wall. The examination was performed on August 31, 2024, using an abdominal non-contrast CT scan.

**Figure 2:**
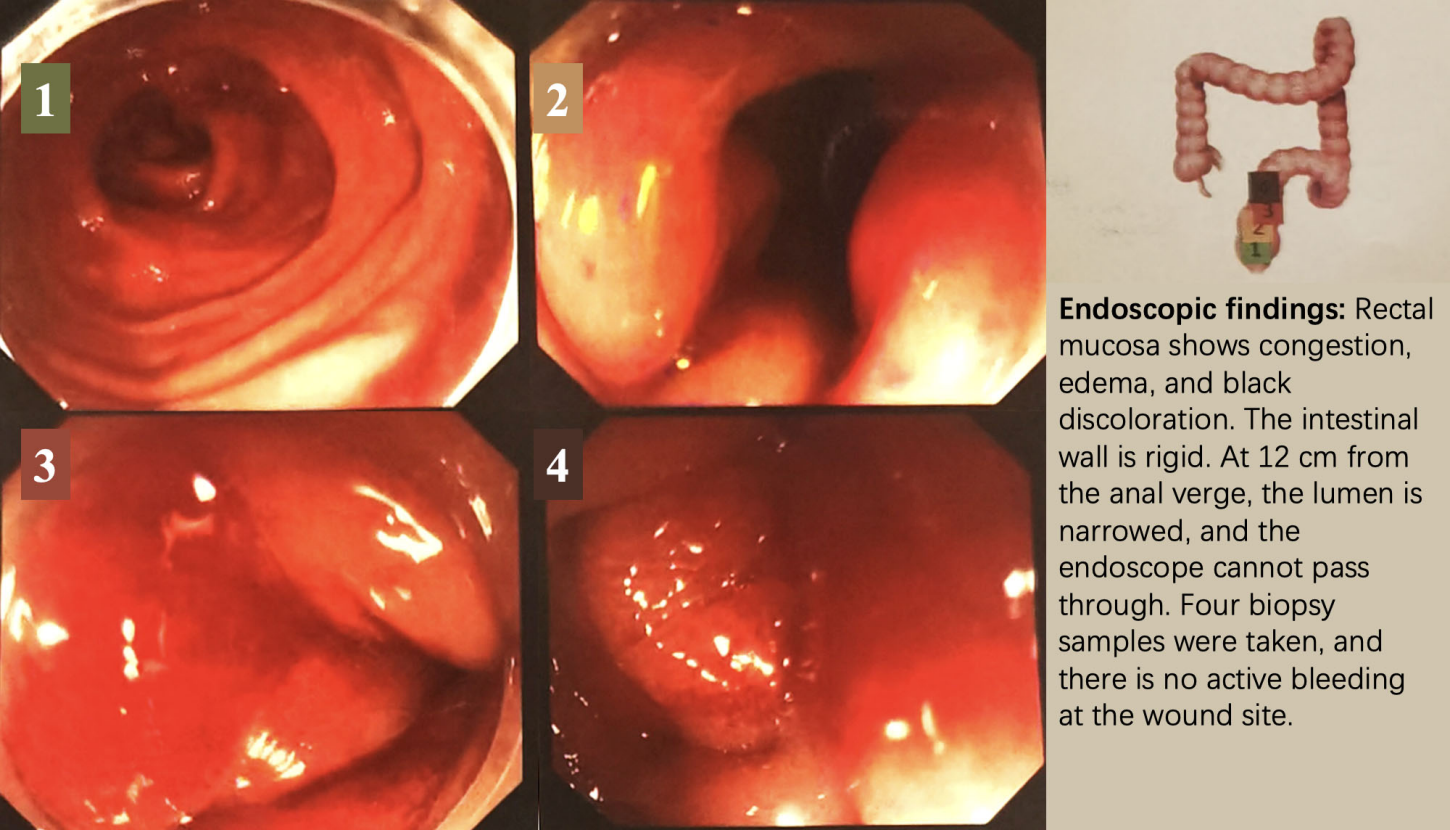
Colonoscopy findings. The colonoscopy images illustrate the position and photographic results of the examination, with different colors corresponding to different areas of the colon examined. The procedure was conducted on September 12, 2024, using a painless colonoscopy technique.

In August 2024, seven years after initial diagnosis, the patient presented with changes in bowel habits and abdominal distention. CT imaging revealed rectal wall thickening (**Figure 1**), and colonoscopy revealed mucosal changes with stenosis 12 cm from the anal verge (**Figure 2**). Biopsy confirmed metastatic breast cancer with immunohistochemistry showing (-1 slice) P53 (mutant type), CK (+), INSM1 (-), Ki67 (50%). (-2 slices) P53 (mutant type), Rb (+), CK (+), CD56 (-), Syn (-), СgА (-), GATA3 (+), CDX2 (-), ER (-), PR (-), Her-2 (1+), GCDFP15 (-), Ki67 50%, SSTR2 (-), INSM1 (-). These findings confirmed that the rectal lesion was a metastasis from the primary breast cancer.

**Figure 3:**
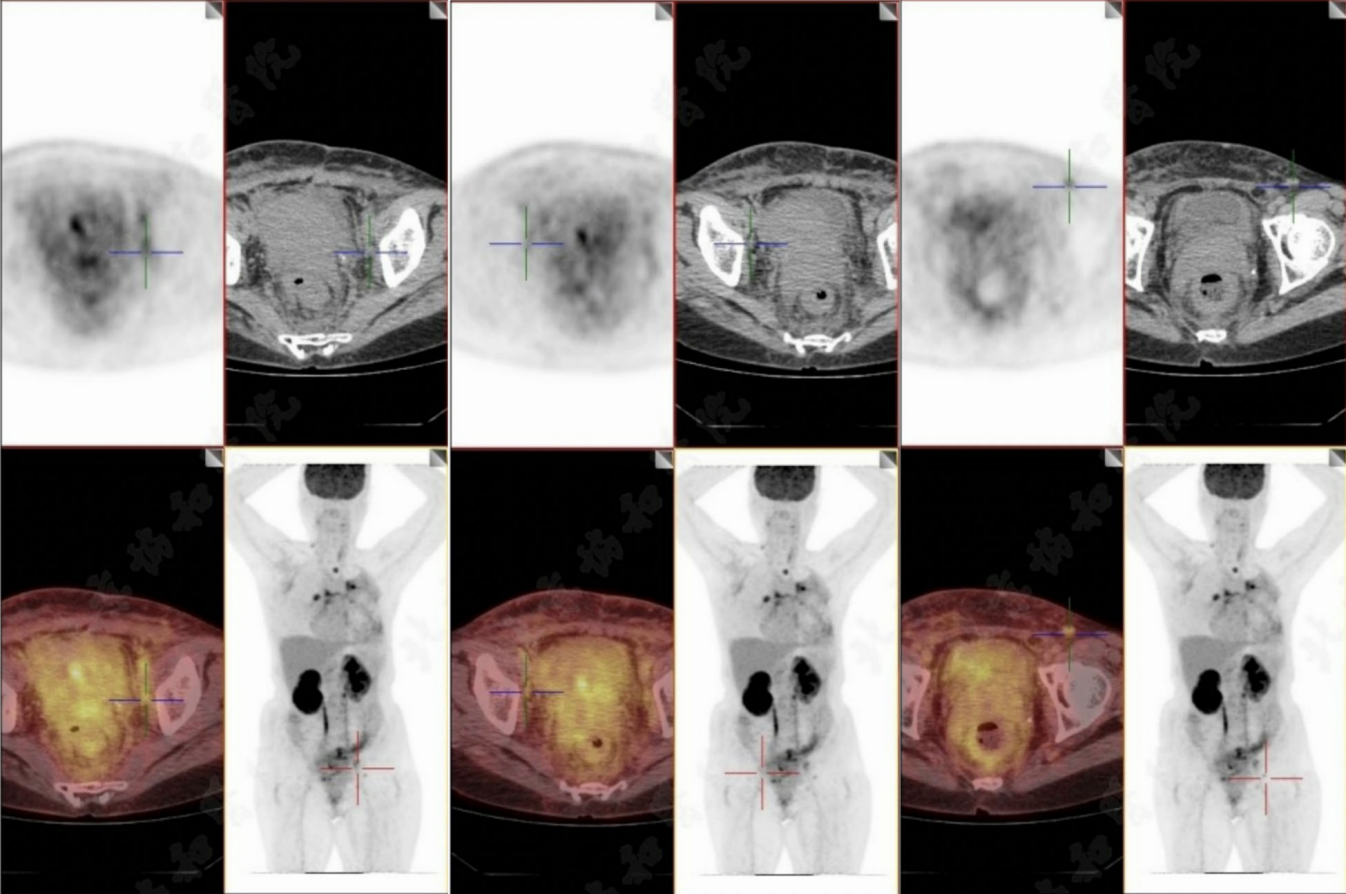
PET-CT imaging of abdominal area. PET-CT scan conducted on September 26, 2024, reveals multiple lymph nodes with increased radioactive uptake located around the abdominal aorta, near the bilateral iliac vessels, and in both inguinal regions. The largest lymph node, measuring approximately 1.1 cm in short diameter, demonstrates a maximum standardized uptake value (SUVmax) of 4.3.

The patient currently has widespread metastatic disease involving multiple sites including bones, lymph nodes, pleura, pericardium, and rectum (**Figure 3**). An intestinal stent was placed to relieve obstruction.

October 2024, the patient’s pericardial effusion has also worsened. After drainage, dyspnea improved slightly. The patient’s general condition remains poor. Our outpatient department recommends intensifying supportive symptomatic treatment. If the patient’s general condition improves, subsequent treatment options may include erlotinib, chemotherapy, or ADC drugs. The patient’s family has requested discharge to seek palliative care.

## Discussion

### Epidemiology and patterns of metastasis

While systemic metastasis of breast cancer (BC) predominantly involves the bones, lungs, liver, and brain, gastrointestinal (GI) tract involvement remains uncommon. A recent comprehensive review revealed that intestinal metastases from BC are detected in less than 5% of patients (3). Maekawa et al. reported that, in a cohort of 980 metastatic BC patients, only 0.5% exhibited GI tract metastases, with rectal involvement being particularly rare (7). Other cohort and case series studies report similarly low incidence rates (8, 9). These data emphasize the rarity of GI metastasis, particularly rectal involvement, which can contribute to underdiagnosis, especially when the clinical presentation is non-specific.

### Diagnostic challenges and pathological confirmation

The clinical symptoms of GI metastasis from BC—such as bowel obstruction, changes in bowel habits, or nonspecific abdominal discomfort—often overlap with those of primary GI malignancies (10, 11). Given these diagnostic challenges, histopathology alone may be insufficient. Immunohistochemical (IHC) profiling is critical for accurate diagnosis. In several reported cases, IHC markers like GATA3 and GCDFP-15, combined with the absence of intestinal markers such as CDX2 or CK20, were pivotal in confirming the breast cancer origin (8, 10). Furthermore, as metastatic breast cancer often undergoes clonal evolution, comparing the IHC profiles of the metastatic site with the original primary tumor is essential for tailoring subsequent therapy (7, 8, 12). This underscores the necessity for a high index of suspicion and a comprehensive IHC evaluation, particularly for markers such as GATA3, which are crucial for confirming breast cancer metastasis (13, 14). Notably, the presence of GI metastases is generally indicative of advanced systemic disease, associated with a poor prognosis (15).

### Patterns of metastasis — which GI sites, latency, subtypes

A review focused on lower GI tract metastases (including the colon, rectum, and small bowel) highlights that, while rare, such cases have been increasingly reported (3, 12). Although invasive lobular carcinoma (ILC) has traditionally been associated with a higher propensity for GI metastasis due to its diffuse growth and loss of E-cadherin, our case demonstrates that invasive ductal carcinoma (IDC) can also metastasize to the GI tract (8, 11, 16). This finding is crucial, as it underscores that clinicians should remain vigilant for GI/rectal metastasis in IDC cases as well. The latency between the initial breast cancer diagnosis and the appearance of GI metastasis can span several years, with some case reports documenting a 5–10-year gap (10, 17).

### Prognosis and treatment outcomes

GI metastases from BC typically signal advanced systemic disease and are associated with a poor prognosis. Median survival following the detection of GI metastasis is often limited, with some series reporting a median survival of approximately one year (3, 10, 11) Given the systemic nature of the disease, systemic therapy remains the cornerstone of treatment. Endoscopic or surgical interventions, such as stent placement, are generally reserved for palliative purposes, particularly in cases of obstruction, bleeding, or severe symptoms (3, 8, 11). Although most data are derived from retrospective case series and reports, these findings emphasize the importance of individualized treatment plans based on receptor status, patient performance status, symptoms, and previous therapies (8).

### Mechanisms of resistance and therapeutic considerations

In hormone receptor-positive (HR+) metastatic breast cancer, acquired resistance to endocrine therapy is frequently driven by mutations in ESR1 (18, 19). ESR1 mutations are recognized as a major mechanism of resistance in metastatic disease, particularly in HR+/HER2– breast cancer. The introduction of CDK4/6 inhibitors (CDK4/6i) has significantly improved the standard of care for this subtype; however, resistance eventually develops (20, 21). Studies on CDK4/6i resistance have identified several molecular alterations, including dysregulation of cell cycle regulators (e.g., RB1 loss, activation of the Cyclin E–CDK2 axis), reactivation of alternative mitogenic pathways (such as PI3K/AKT/mTOR), and possible epigenetic reprogramming (22). These alterations contribute to a shift toward estrogen-independent, more aggressive tumor clones (23–26).

In the case of our patient, the evolution from ER-positive IDC to ER-negative metastatic disease may have selected for subclones with increased invasive capacity, survival advantage, and the ability to colonize uncommon sites like the rectum. While direct evidence linking these molecular changes to rectal tropism is lacking, the convergence of receptor loss, cell-cycle deregulation, and therapy resistance provides a plausible biological explanation for the observed metastatic pattern. Once hormone receptor expression is lost, endocrine therapy becomes ineffective, and systemic chemotherapy remains the primary treatment option. Additionally, as our understanding of resistance mechanisms advances, novel therapeutic strategies targeting downstream pathways (e.g., PI3K/mTOR inhibitors) may offer new treatment avenues for future cases (20, 23). In the context of GI obstruction due to metastatic lesions, local palliative interventions, such as stent placement, are crucial for symptomatic relief and maintaining quality of life. A multidisciplinary approach, involving oncologists, gastroenterologists, and surgeons, is essential for managing such complex metastatic disease.

## Conclusion

This case highlights the importance of considering rectal metastasis in the differential diagnosis of gastrointestinal symptoms in breast cancer patients, even many years after initial diagnosis. It emphasizes the critical role of comprehensive pathological evaluation and IHC analysis in confirming metastatic breast cancer. Given the molecular mechanisms underlying tumor progression, it is essential to reassess the tumor’s biological characteristics throughout treatment to guide appropriate therapeutic strategies.

## Data Availability

The original contributions presented in the study are included in the article/supplementary material. Further inquiries can be directed to the corresponding author.

## References

[1] Siegel RL Miller KD Jemal A . Cancer statistics, 2020. CA Cancer J Clin. (2020) 70:7–30. doi: 10.3322/caac.21590, PMID: 31912902

[2] McLemore EC Pockaj BA Reynolds C Gray RJ Hernandez JL Grant CS . Breast cancer: presentation and intervention in women with gastrointestinal metastasis and carcinomatosis. Ann Surg Oncol. (2005) 12:886–94. doi: 10.1245/ASO.2005.03.030, PMID: 16177864

[3] Bolzacchini E Nigro O Inversini D Giordano M Maconi G . Intestinal metastasis from breast cancer: Presentation, treatment and survival from a systematic literature review. World J Clin Oncol. (2021) 12:382–92. doi: 10.5306/wjco.v12.i5.382, PMID: 34131569 PMC8173325

[4] Harris M Howell A Chrissohou M Swindell RI Hudson M Sellwood RA . A comparison of the metastatic pattern of infiltrating lobular carcinoma and infiltrating duct carcinoma of the breast. Br J Cancer. (1984) 50:23–30. doi: 10.1038/bjc.1984.135, PMID: 6331484 PMC1976917

[5] Borst MJ Ingold JA . Metastatic patterns of invasive lobular versus invasive ductal carcinoma of the breast. Surgery. (1993) 114:637–41., PMID: 8211676

[6] Selves J Long-Mira E Mathieu MC Rochaix P Ilié M . Immunohistochemistry for diagnosis of metastatic carcinomas of unknown primary site. Cancers (Basel). (2018) 10:108. doi: 10.3390/cancers10040108, PMID: 29621151 PMC5923363

[7] Ambroggi M Stroppa EM Mordenti P Biasini C Zangrandi A Michieletti E . Metastatic breast cancer to the gastrointestinal tract: report of five cases and review of the literature. Int J Breast Cancer. (2012) 2012:439023. doi: 10.1155/2012/439023, PMID: 23091732 PMC3471430

[8] Zhong C Fang X Zhu L Li D Tang J Yuan Y . Report of two cases and a systematic review of breast cancer with gastrointestinal metastasis. Turk J Gastroenterol. (2019) 30:997–1000. doi: 10.5152/tjg.2019.18649, PMID: 31767559 PMC6883985

[9] Nazareno J Taves D Preiksaitis HG . Metastatic breast cancer to the gastrointestinal tract: a case series and review of the literature. World J Gastroenterol. (2006) 12:6219–24. doi: 10.3748/wjg.v12.i38.6219, PMID: 17036400 PMC4088122

[10] Jones GE Strauss DC Forshaw MJ Deere H Mahedeva U Mason RC . Breast cancer metastasis to the stomach may mimic primary gastric cancer: report of two cases and review of literature. World J Surg Oncol. (2007) 5:75. doi: 10.1186/1477-7819-5-75, PMID: 17620117 PMC1937002

[11] Villa Guzmán JC Espinosa J Cervera R Delgado M Patón R Cordero García JM . Gastric and colon metastasis from breast cancer: case report, review of the literature, and possible underlying mechanisms. Breast Cancer (Dove Med Press). (2017) 9:1–7. doi: 10.2147/bctt.S79506, PMID: 28096693 PMC5207330

[12] Da Cunha T Saleh SA Dharan M . Breast cancer metastasizing to the lower gastrointestinal tract (the small bowel and colon): A case presentation and comprehensive review of the literature. Oncol Adv. (2024) 2:91–9. doi: 10.14218/OnA.2024.00001

[13] Sasaki E Tsunoda N Hatanaka Y Mori N Iwata H Yatabe Y . Breast-specific expression of MGB1/mammaglobin: an examination of 480 tumors from various organs and clinicopathological analysis of MGB1-positive breast cancers. Mod Pathol. (2007) 20:208–14. doi: 10.1038/modpathol.3800731, PMID: 17192791

[14] Miettinen M McCue PA Sarlomo-Rikala M Rys J Czapiewski P Wazny K . GATA3: a multispecific but potentially useful marker in surgical pathology: a systematic analysis of 2500 epithelial and nonepithelial tumors. Am J Surg Pathol. (2014) 38:13–22. doi: 10.1097/PAS.0b013e3182a0218f, PMID: 24145643 PMC3991431

[15] Taal BG Peterse H Boot H . Clinical presentation, endoscopic features, and treatment of gastric metastases from breast carcinoma. Cancer. (2000) 89:2214–21. doi: 10.1002/1097-0142(20001201)89:11<2214::AID-CNCR9>3.0.CO;2-D, PMID: 11147591

[16] Bai X Fang C Liu B Huagn J Chen X Xie X . Breast cancer metastases to the thyroid and stomach: A case report. Oncol Lett. (2023) 26:386. doi: 10.3892/ol.2023.13972, PMID: 37559588 PMC10407839

[17] Zhao Q Dou L Feng X Wang G He S . Breast cancer metastases to the stomach with endoscopic submucosal dissection: a case report and literature study. Front Oncol. (2025) 15. doi: 10.3389/fonc.2025.1573163, PMID: 40475018 PMC12137079

[18] Brett JO Spring LM Bardia A Wander SA . ESR1 mutation as an emerging clinical biomarker in metastatic hormone receptor-positive breast cancer. Breast Cancer Res. (2021) 23:85. doi: 10.1186/s13058-021-01462-3, PMID: 34392831 PMC8365900

[19] Pejerrey SM Dustin D Kim JA Gu G Rechoum Y Fuqua SAW . The impact of ESR1 mutations on the treatment of metastatic breast cancer. Horm Cancer. (2018) 9:215–28. doi: 10.1007/s12672-017-0306-5, PMID: 29736566 PMC9680193

[20] Mittal A Molto Valiente C Tamimi F Schlam I Sammons S Tolaney SM . Filling the gap after CDK4/6 inhibitors: novel endocrine and biologic treatment options for metastatic hormone receptor positive breast cancer. Cancers (Basel). (2023) 15:2015. doi: 10.3390/cancers15072015, PMID: 37046675 PMC10093251

[21] Chang CM Lam HYP . Mechanism of CDK4/6 inhibitor resistance in hormone receptor-positive breast cancer and alternative treatment strategies. Anticancer Res. (2023) 43:5283–98. doi: 10.21873/anticanres.16732, PMID: 38030174

[22] Wander SA Cohen O Gong X Johnson GN Buendia-Buendia JE Lloyd MR . The genomic landscape of intrinsic and acquired resistance to cyclin-dependent kinase 4/6 inhibitors in patients with hormone receptor-positive metastatic breast cancer. Cancer Discov. (2020) 10:1174–93. doi: 10.1158/2159-8290.CD-19-1390, PMID: 32404308 PMC8815415

[23] Abdullah HMA Chennapragada SS Singh R Zeidalkilani JMJ Kesireddy M . Precision therapy in metastatic breast cancer: the current landscape of molecular alteration-based therapies. Transl Breast Cancer Res. (2025) 6:24. doi: 10.21037/tbcr-25-11, PMID: 40756958 PMC12314682

[24] Chaudhary N Chibly AM Collier A Martinalbo J Perez-Moreno P Moore HM . CDK4/6i-treated HR+/HER2- breast cancer tumors show higher ESR1 mutation prevalence and more altered genomic landscape. NPJ Breast Cancer. (2024) 10:15. doi: 10.1038/s41523-024-00617-7, PMID: 38388477 PMC10883990

[25] Kan Z Wen J Bonato V Webster J Yang W Ivanov V . Real-world clinical multi-omics analyses reveal bifurcation of ER-independent and ER-dependent drug resistance to CDK4/6 inhibitors. Nat Commun. (2025) 16:932. doi: 10.1038/s41467-025-55914-x, PMID: 39843429 PMC11754447

[26] Zhou FH Downton T Freelander A Hurwitz J Caldon CE Lim E . CDK4/6 inhibitor resistance in estrogen receptor positive breast cancer, a 2023 perspective. Front Cell Dev Biol. (2023) 11. doi: 10.3389/fcell.2023.1148792, PMID: 37035239 PMC10073728

